# Adenomyosis, Infertility and Maternal–Fetal Outcomes: Diagnostic and Therapeutic Strategies Across Disease Phenotype—A Narrative Review

**DOI:** 10.3390/jcm15176722

**Published:** 2026-08-29

**Authors:** Francesco Giuseppe Martire, Eugenia Costantini, Ilaria Ianes, Claudia d’Abate, Giuseppe Sorrenti, Maria De Bonis, Lucia Lazzeri, Errico Zupi

**Affiliations:** 1 Department of Molecular and Developmental Medicine, Obstetrics and Gynecological Clinic, University of Siena, 53100 Siena, Italy; francescogmartire@libero.it (F.G.M.); eugenia.costantini22@gmail.com (E.C.); ilaria.ianes@gmail.com (I.I.); claudiadabate94@gmail.com (C.d.); mariadebonis@gmail.com (M.D.B.); zupi@unisi.it (E.Z.); 2 Department of Gynecology, San Carlo Nancy Hospital, 00165 Rome, Italy; giuseppe.sorrenti@gmail.com

**Keywords:** adenomyosis, infertility, maternal-fetal outcomes, phenotype, tailored management

## Abstract

Adenomyosis is a chronic estrogen-dependent disorder that may adversely affect reproductive health. Increasing evidence suggests that its reproductive and obstetric consequences may vary according to disease phenotype, including focal versus diffuse forms and inner versus outer myometrial localization. This narrative review summarizes current evidence on adenomyosis, infertility, recurrent pregnancy loss, and maternal–fetal outcomes, with particular attention to phenotype-specific clinical implications. MEDLINE and Scopus were searched for English-language studies from database inception through March 2026, and the selected evidence was qualitatively synthesized according to the main clinical topics. Available evidence indicates that adenomyosis is associated with impaired fertility through defective endometrial receptivity, progesterone resistance, abnormal uterine peristalsis, chronic inflammation, and immune dysregulation. Adenomyosis has also been associated with miscarriage and adverse obstetric outcomes, including preterm birth, hypertensive disorders, placental dysfunction, fetal growth restriction, and postpartum hemorrhage. Emerging data suggest distinct profiles, with internal adenomyosis more closely related to implantation failure, recurrent pregnancy loss, and placental complications, whereas external adenomyosis is more frequently associated with endometriosis and primary infertility. However, phenotype-specific associations require further validation. Accurate phenotypic characterization may improve reproductive counseling and individualized management. Prospective studies using standardized diagnostic criteria are needed to confirm phenotype-specific risks and develop targeted therapeutic and obstetric strategies.

## 1. Introduction

Adenomyosis is a benign, chronic, estrogen-related condition that has a significant impact on women’s health [1]. Although it was first described in 1860 [2], until very recently it was always considered a condition affecting women of perimenopausal age, often multiparous, who, having completed their reproductive years, underwent radical hysterectomy to treat debilitating symptoms such as pelvic pain and/or heavy menstrual bleeding [3]. Over the last decade, awareness of this condition has grown, and it is now widely accepted that adenomyosis has an early onset, occurring after menarche, with painful symptoms and heavy periods that are present right from the very first menstrual cycles [4,5]. What manifests later, however, is the impact on fertility, and even later still is the impact on pregnancy [6]. With regard to this condition, the latest evidence suggests that it is not so much the severity of the disease—whether mild, moderate or severe—that worsens reproductive and obstetric outcomes, but rather the type of disease (focal or diffuse) and, even more so, its location (internal myometrium or external myometrium) that impacts these outcomes [7]. This idea is also linked to the various pathogenetic theories of the disease, in which the Tissue Injury and Repair (TIAR) mechanism posits the primary cause within the junctional zone and explains the adenomyosis of the inner myometrium with outward migration [8], whilst the Coelomic Epithelium Theory suggests that the disease originates from concomitant deep infiltrating endometriosis, which progresses from the outside inwards assuming different symptoms [9]. Based on this premise, early diagnosis—not only of the presence of the disease but also of its type and location—can become fundamental in the management of these patients, enabling the selection of the most appropriate preventive and/or therapeutic strategies for both fertility treatment and pregnancy management [10].

The primary aim of this narrative review is to examine the reproductive and obstetric implications of adenomyosis according to disease phenotype and anatomical localization, with particular attention to infertility, recurrent pregnancy loss, and maternal–fetal outcomes. We also discuss the potential implications of phenotypic characterization for diagnosis, reproductive counseling, therapeutic management, and pregnancy surveillance.

## 2. Materials and Methods

We performed an electronic literature search using the MEDLINE database and Scopus to identify English-language articles investigating adenomyosis, infertility, recurrent pregnancy loss (RPL), and maternal–fetal outcomes published from database inception through March 2026. A combination of the following keywords and Medical Subject Headings (MeSH) terms was used to identify relevant studies: “Adenomyosis”, “Infertility”, “Maternal–foetal outcomes”, “Miscarriage”, “Pregnancy”, and “Recurrent Pregnancy Loss”. Original research articles, including randomized and non-randomized clinical trials, prospective and retrospective observational studies, case–control studies, as well as systematic reviews, meta-analyses, and relevant review articles, were considered. Attention was given to studies reporting data according to adenomyosis phenotype, including disease localization within the inner (junctional zone) or outer myometrium, as well as focal and diffuse forms, and their association with reproductive and obstetric outcomes. The literature search and identification of relevant studies were conducted by two authors (F.G.M. and L.L.). Given the narrative nature of this review, the selected evidence was qualitatively synthesized according to the main clinical topics addressed. The literature search was intended to support a clinically oriented narrative synthesis rather than a systematic evidence review. The selected studies were grouped according to the main clinical topics addressed, including infertility, recurrent pregnancy loss, maternal–foetal outcomes, and therapeutic strategies, with particular emphasis on the potential impact of adenomyosis phenotype and lesion localization on reproductive and obstetric outcomes.

## 3. Adenomyosis: General Aspects

### 3.1. Epidemiology

The true prevalence of adenomyosis remains difficult to determine accurately, as most of the available data are derived from histopathological diagnoses [11]. Over the past 50 years, the reported prevalence among women undergoing hysterectomy has ranged from 8.8% to 61.5%, mainly due to the lack of standardized histopathological criteria, differences in tissue sampling, and variability among pathologists [12,13].

Histopathological diagnosis is particularly challenging in minimal disease and depends on the extent of tissue sampling. Bergholt et al. reported an adenomyosis prevalence ranging from 10.0% to 18.2% depending on the histopathological diagnostic criteria applied [14]. Earlier, Bird et al. demonstrated that prevalence estimates could increase from 31% to 61.5% with more extensive histological assessment and the inclusion of superficial disease [15]. Moreover, no universally accepted histological criteria currently exist. Several cut-offs have been proposed, ranging from approximately 2.5 to 8 mm from the endometrial–myometrial junction or according to fractions of uterine wall thickness [11].

The 2.5 mm threshold is the most commonly adopted, although it remains arbitrary and may underestimate milder but clinically relevant disease [16,17].

The historical reliance on histopathological diagnosis has likely contributed to the perception of adenomyosis as a condition predominantly affecting older women [18]. The first ultrasound-based prevalence study reported adenomyosis in 20.9% of women undergoing transvaginal ultrasound (TVUS) for gynecological symptoms [19].

Similarly, an Italian study of 156 nulliparous women aged 18–30 years without major gynecological risk factors found a prevalence of 34%, suggesting that adenomyosis may also affect young and asymptomatic women [20]. As with histological diagnosis, no universally accepted imaging criteria are currently available, potentially influencing prevalence estimates. Although TVUS is operator-dependent and may be affected by hormonal treatment [21], it remains a highly effective diagnostic tool [11]. Meta-analyses have demonstrated that TVUS and magnetic resonance imaging (MRI) show comparable diagnostic performance for adenomyosis, with no significant differences in sensitivity or specificity [22,23].

The prevalence of adenomyosis appears to be even higher in specific clinical settings. Among women with endometriosis and infertility, reported rates range from 35% to 79% [24,25,26], while in those with endometriosis-associated pelvic pain prevalence ranges from 38% to 87% [9]. In women with leiomyomas, reported prevalence varies between 16% and 62%, depending on the study population and diagnostic setting [27].

Estimating the incidence of adenomyosis is equally challenging. Population-based studies from Italy and the United States have likely underestimated the true incidence of the disease because of non-standardized diagnostic criteria and the historical reliance on hysterectomy-based diagnosis [28,29]. The absence of screening programs and the lack of universally accepted diagnostic criteria further contribute to underestimation.

Therefore, improved standardization of both histological and imaging diagnostic criteria represents a crucial step toward accurately defining the true prevalence and clinical impact of adenomyosis [30].

### 3.2. Pathogenesis

Although the pathogenesis of adenomyosis remains incompletely understood, several theories have been proposed to explain its origin. The mechanistic evidence discussed in this section derives mainly from human tissue, cellular and molecular studies, whereas the subsequent sections addressing reproductive and obstetric outcomes primarily focus on clinical studies in women. The two main hypotheses are the Tissue Injury and Repair theory and the Coelomic Epithelium Theory.

- **Tissue Injury and Repair (TIAR)**

According to the most widely accepted hypothesis, basal endometrial tissue invades the myometrium through disruption of the endometrial–myometrial junctional zone (JZ) caused by repeated tissue injury [31]. Local and ovarian-derived estrogens create a hyperestrogenic environment that increases uterine contractility and mechanical strain, further damaging the JZ and promoting endometrial invasion into the myometrium [31]. The invasive capacity of endometrial cells has been associated with increased expression of genes involved in cell motility and tissue remodeling, processes that may be promoted by estrogenic and inflammatory stimuli [32,33,34,35].

- **Coelomic Epithelium Theory**

An alternative hypothesis suggests that adenomyotic lesions arise de novo, likely due to the differentiation of displaced embryonic Müllerian remnants into endometrial-like tissue [36]. The presence of Müllerian-derived structures in fetal organs supports this theory, although direct experimental confirmation is still lacking [36,37]. Endometrial stem and progenitor cells may also contribute to lesion development. Similar to Sampson’s theory of endometriosis, these cells may be transported to ectopic uterine sites and promote adenomyotic lesion formation [34,38,39].

In addition to these two theories, there are other factors that play a role in pathogenesis, which may become therapeutic targets [40]:

- **Impact of estrogens on endometrial cells**

Adenomyosis is considered an estrogen-dependent disease. Estrogens promote endometrial cell proliferation, epithelial–mesenchymal transition (EMT), angiogenesis, and tissue invasion, while also influencing myometrial function through alterations in estrogen receptor expression and signaling pathways [41,42,43,44,45,46].

- **Role of the immune system**

Immune dysregulation appears to play a central role in adenomyosis. Increased numbers of macrophages, uterine natural killer cells, and altered T-cell populations have been identified in the endometrium of affected women [47,48,49,50,51].

According to the TIAR hypothesis, hyperestrogenism promotes inflammatory cell recruitment, establishing a chronic inflammatory microenvironment that further enhances estrogen production and tissue remodeling [31]. Altered estrogen receptor expression, local estrogen synthesis, and immune activation may interact to sustain disease progression [34,52,53,54,55,56].

- **Genetic predisposition theory**

Growing evidence suggests that genetic and epigenetic factors contribute to adenomyosis susceptibility. Mutations in genes involved in cell proliferation and differentiation, including KRAS and PIK3CA, may facilitate endometrial invasion into the myometrium [9,57]. In addition, epigenetic mechanisms such as DNA methylation and histone modifications may alter the expression of genes involved in hormonal signaling and endometrial growth, contributing to disease development and progression [58,59].

- **Emerging molecular and cellular insights into adenomyosis pathogenesis**

While hormonal, inflammatory, and genetic mechanisms represent key components of adenomyosis pathogenesis, recent advances in molecular profiling technologies have revealed a higher degree of cellular complexity and heterogeneity than previously recognized. Recent advances in high-resolution molecular profiling have expanded the understanding of adenomyosis [60].

Transcriptomic analyses of eutopic endometrium have identified altered molecular signatures associated with impaired implantation, defective endometrial receptivity, and pregnancy complications in women with adenomyosis [61].

Single-cell RNA sequencing and spatial transcriptomic approaches have started to reveal distinct cellular populations within adenomyotic lesions and the surrounding myometrium, identifying transcriptionally heterogeneous stromal fibroblast subsets, epithelial populations with altered differentiation programs, and immune cell clusters involved in tissue remodeling and inflammation. In particular, stromal fibroblast heterogeneity appears to represent a key determinant of disease phenotype, with specific fibroblast subpopulations exhibiting pro-fibrotic, inflammatory, and extracellular matrix-remodeling signatures [62].

The interaction between stromal cells, epithelial cells, and immune components may contribute to the establishment of a permissive microenvironment characterized by altered tissue architecture, fibrosis, and impaired endometrial–myometrial communication. Cell–cell communication analyses have further highlighted the role of paracrine signaling pathways, including TGF-β, IL-6/JAK/STAT, TNF-related pathways, and chemokine signaling, in sustaining inflammation and promoting pathological remodeling [63].

Moreover, increasing evidence suggests that macrophage polarization and EMT may contribute to adenomyosis progression. Alterations in macrophage polarization profiles may contribute to angiogenesis, fibrosis, and maladaptive tissue remodeling in adenomyosis, whereas EMT-related processes may enhance epithelial plasticity, cellular migration, and extracellular matrix deposition [64].

Collectively, these emerging molecular insights provide a mechanistic link between cellular reprogramming, altered uterine microenvironment, impaired endometrial receptivity, and adverse reproductive outcomes, offering new perspectives for understanding the heterogeneous clinical manifestations of adenomyosis and for the development of phenotype-oriented therapeutic strategies.

To summarize the main molecular and cellular mechanisms implicated in adenomyosis pathogenesis, the major pathways, key mediators, and potential therapeutic implications are summarized in Table 1.

### 3.3. Symptoms

Adenomyosis is a heterogeneous gynecological condition characterized by a wide spectrum of clinical manifestations, ranging from asymptomatic cases to severe symptomatic disease. The most common symptoms include dysmenorrhea, heavy menstrual bleeding (HMB), chronic pelvic pain, dyspareunia, and infertility, although clinical presentation is highly variable and frequently overlaps with other gynecological disorders such as endometriosis and uterine fibroids, which commonly coexist and may act as confounding factors. Approximately 30% of affected women may be asymptomatic, highlighting the importance of imaging for diagnosis [11].

Among symptomatic patients, dysmenorrhea and HMB are the most frequently reported complaints, particularly in younger women. Indeed, adenomyosis may already be present during adolescence and early reproductive age and has been significantly associated with HMB and coexisting dysmenorrhea [65,66]. Clinical expressions may vary according to age and disease phenotype. Younger women more frequently report pelvic pain and dysmenorrhea, whereas HMB becomes more prominent with increasing age [4]. In addition, internal adenomyosis is more frequently associated with abnormal uterine bleeding, while external forms are often linked to coexisting endometriosis [67,68].

Beyond pain and bleeding symptoms, adenomyosis is increasingly recognized as a contributor to adverse reproductive outcomes. The disease has been associated with infertility, recurrent pregnancy loss, and reduced live birth rates, particularly in more severe or internal forms [69]. However, the relationship between disease severity, clinical manifestations, and reproductive outcomes remains complex and not fully understood.

### 3.4. Diagnosis

Adenomyosis should be suspected in the presence of suggestive clinical features, including menstrual and gynecological history, although it may be identified in asymptomatic patients. The first-line diagnostic tool is TVUS, performed using both 2D and 3D techniques. Meta-analytic data indicate pooled sensitivity and specificity of approximately 78% and 78% for TVUS and 78% and 88% for MRI, respectively [21]. A head-to-head meta-analysis comparing both modalities in the same populations reported similar diagnostic performance, with sensitivity and specificity of 75% and 81% for TVUS and 69% and 80% for MRI, respectively, although substantial heterogeneity was observed across studies [23]. Therefore, the choice between TVUS and MRI depends on availability, operator expertise, and the clinical setting. Typical MRI features supporting the diagnosis include focal or diffuse widening of the junctional zone and the presence of simple or hemorrhagic myometrial cysts [70].

TVUS evaluation should be systematic and include assessment of uterine morphology, myometrial structure, and the junctional zone. The introduction of the revised Morphological Uterus Sonographic Assessment (MUSA 2022) consensus [71], has further standardized the sonographic diagnosis of adenomyosis by distinguishing between direct and indirect ultrasound features. The presence of at least one direct sign—such as myometrial cysts, hyperechogenic islands, or echogenic subendometrial lines or buds—is considered sufficient for diagnosis, as these reflect ectopic endometrial tissue within the myometrium. In contrast, indirect signs, including globular uterus, asymmetrical myometrial thickening, fan-shaped shadowing, translesional vascularity, and irregular or interrupted JZ, are secondary manifestations and, when present alone, are not diagnostic [71]. Direct features generally show high specificity but variable and relatively limited sensitivity, whereas indirect features may also occur in the absence of adenomyosis and should therefore be interpreted in the context of the overall sonographic pattern [71]. Beyond diagnosis, a comprehensive characterization of adenomyosis is essential. TVUS allows classification of adenomyosis into diffuse, focal, and adenomyoma forms, while also assessing the depth and anatomical location of myometrial involvement [72]. Lesions may involve either the internal myometrium, including the JZ, or the external myometrium, with potentially different clinical implications. Available phenotype-specific evidence suggests that internal adenomyosis (inner-myometrial/JZ involvement) is more closely associated with impaired implantation, recurrent pregnancy loss, and placental dysfunction-related obstetric complications. In contrast, external adenomyosis is more frequently associated with coexisting endometriosis and primary infertility. However, these phenotype-specific associations remain based on emerging and heterogeneous evidence and require further validation [7,67,68,69,73,74,75]. The severity of adenomyosis can also be graded according to the extent of myometrial involvement, typically defined as mild (<25%), moderate (25–50%), or severe (>50%). Importantly, although some correlations exist—such as the association between diffuse disease and symptoms like dysmenorrhea or heavy menstrual bleeding—the severity of imaging findings does not always correspond to symptom intensity, likely due to the frequent coexistence of other conditions such as endometriosis [76]. In selected clinical settings, a comprehensive diagnostic work-up may also benefit from hysteroscopic evaluation. Although hysteroscopy is not a primary diagnostic tool for adenomyosis, it may provide complementary information in infertile patients by identifying and, when indicated, treating concomitant intrauterine abnormalities that could impair reproductive outcomes. However, its routine use remains controversial and should be individualized according to clinical indications [77].

Early imaging surveillance may also have important implications for disease progression and reproductive counseling. In this regard, Martire et al. demonstrated that ultrasound follow-up in young women with severe dysmenorrhea can predict the early onset of endometriosis, supporting the concept that timely imaging assessment may facilitate earlier recognition of progressive gynecological disorders and improve long-term clinical management [78].

For these reasons, it is crucial not only to establish the presence of adenomyosis but also to describe its phenotype in detail, including type (diffuse, focal, or adenomyoma), location (internal vs. external myometrium), uterine distribution, and severity. A more precise phenotypic classification may allow, in the future, a better correlation between disease characteristics, clinical presentation, reproductive outcomes, and response to treatment [79].

Although imaging techniques remain the cornerstone of adenomyosis diagnosis, increasing interest has focused on molecular biomarkers that may improve disease characterization and prognostic assessment. Circulating microRNAs [80], extracellular-vesicle [81] and epigenetic signatures in circulating cell-free DNA [82] have been investigated as potential non-invasive biomarkers due to their involvement in gene regulation, cell communication, and inflammatory responses. In addition, circulating inflammatory cytokines, proteomic signatures, and metabolomic profiles have been explored to identify molecular patterns associated with immune activation, fibrosis, hormonal imbalance, and altered endometrial function [83]. However, current evidence remains limited and heterogeneous, and these biomarkers require further validation before their clinical application. Future integration of molecular biomarkers with imaging findings and clinical phenotypes may support a more personalized approach to adenomyosis diagnosis and management.

### 3.5. Infertility

Investigating the impact of adenomyosis on female fertility is particularly challenging due to its frequent co-existence with other gynecological conditions—such as endometriosis and uterine fibroids, which often act as confounding factors. However, recent evidence consistently associates adenomyosis with a significant reduction in reproductive potential across several dimensions.

Adenomyosis exerts a documented negative effect on both natural conception and Assisted Reproductive Technology (ART) outcomes. Specifically, in a systematic review and meta-analysis including 1865 women, Vercellini et al. reported a 28% reduction in the likelihood of clinical pregnancy following IVF/ICSI compared with women without adenomyosis (RR 0.72, 95% CI 0.55–0.95) [84]. Adenomyosis has also been associated with an increased risk of miscarriage following IVF/ICSI (RR 2.12, 95% CI 1.20–3.75) [84]. A subsequent systematic review and meta-analysis reported lower odds of live birth (OR 0.59, 95% CI 0.37–0.92) and higher odds of miscarriage (OR 2.11, 95% CI 1.33–3.33) in women with adenomyosis compared with controls [85]. Although some studies have failed to demonstrate a significant association, leading to partial inconsistency in the literature, the overall trend identifies adenomyosis as a relevant risk factor for reproductive failure. Recent ART studies have further shown that adenomyosis may adversely affect reproductive outcomes even after single euploid embryo transfer, while specific ultrasound-defined adenomyosis features, particularly those involving the inner myometrium, have been associated with reduced live birth rates after IVF/ICSI [86,87].

Furthermore, different phenotypes of adenomyosis have been shown to be associated with distinct reproductive patterns, including different forms of infertility, RPL, and potentially variable outcomes following ART. In particular, patients with external adenomyosis appear to present primary infertility more frequently than those with internal adenomyosis (41.3% vs. 20.7%). Conversely, internal adenomyosis seems to be more strongly associated with RPL and secondary infertility. With regard to ART outcomes, the currently available evidence remains limited, preventing definitive conclusions from being drawn [7,88,89].

The subfertility associated with adenomyosis is thought to result from a combination of anatomical, functional, hormonal, molecular, and immunological alterations, which collectively contribute to the establishment of a hostile uterine environment impairing implantation and pregnancy progression. Numerous mechanisms have been proposed to explain the association between adenomyosis and infertility.

(A) Impaired endometrial receptivity

One of the central mechanisms underlying infertility in adenomyosis is reduced endometrial receptivity. Mounting evidence indicates that alterations in the eutopic endometrium significantly compromise this receptive state, even in the absence of overt structural abnormalities. Adenomyosis is associated with dysregulation of key implantation-related molecules, including integrins (such as αvβ3), leukemia inhibitory factor, and transcription factors such as HOXA10 and HOXA11, which are essential for endometrial differentiation and embryo–endometrium interaction [90]. Wu et al. (2024) highlight that aberrant expression of these molecules leads to a reduction in the “window of implantation” efficiency, thereby impairing embryo attachment and invasion [91]. Additionally, defective decidualization of endometrial stromal cells further compromises implantation success [92].

A hallmark of adenomyosis-associated infertility is progesterone resistance, characterized by altered progesterone receptor expression and epigenetic modifications, such as promoter hypermethylation, resulting in impaired downstream signaling. Consequently, the endometrium fails to undergo adequate decidual transformation, which is essential for embryo implantation and early placentation [93].

(B) Altered Uterine Peristalsis

Alterations in uterine peristalsis represent another key factor contributing to subfertility. In adenomyosis, abnormal myometrial contractility has been demonstrated, including hyperperistalsis, increased contraction amplitude, and dysregulated peristaltic patterns, particularly during the peri-ovulatory phase.

These abnormalities may impair sperm transport, oocyte migration, and embryo positioning within the uterine cavity. Moreover, altered contractility may contribute both to the typical clinical symptoms (such as dysmenorrhea and heavy menstrual bleeding) and to reduced fertility potential [94].

(C) Chronic inflammation and immune dysregulation

The adenomyotic endometrium is characterized by a chronic inflammatory microenvironment, driven by increased levels of pro-inflammatory mediators and an altered immune response, which together impair implantation and pregnancy maintenance [95].

Within this altered microenvironment, macrophage populations undergo significant functional changes, with a predominance of pro-inflammatory phenotypes and dysregulation of tissue-remodeling functions. This imbalance is associated with increased levels of cytokines such as interleukin-6, interleukin-18, and tumor necrosis factor-α, together with alterations in key immune tolerance mediators, including interleukin-10 and transforming growth factor-β. Transforming growth factor-β contributes to fibrosis and promotes epithelial–mesenchymal transition, further exacerbating structural and functional abnormalities of the endometrium [90,92].

Alterations in uterine natural killer cell activity and T-cell subsets further impair maternal immune tolerance. Increased cytotoxicity of natural killer cells and an imbalance between pro-inflammatory T helper 17 cells and regulatory T cells may hinder the establishment of immune acceptance of the embryo, thereby increasing the risk of implantation failure and early pregnancy loss [90].

Collectively, these inflammatory and immune alterations disrupt endometrial function through impaired decidualization, defective vascular remodeling, oxidative stress, fibrosis, and abnormal uterine contractility. These mechanisms reinforce the hostile uterine environment characteristic of adenomyosis and progressively compromise reproductive outcomes [96].

### 3.6. Recurrent Pregnancy Loss

Recurrent pregnancy loss, defined as the occurrence of two or more failed clinical pregnancies, represents a complex and heterogeneous condition in which embryonic, uterine, endocrine, and immunological factors may interact [97]. Although embryonic aneuploidy remains the most frequent cause of miscarriage, particularly in early gestation, increasing evidence suggests that uterine dysfunction plays a central role in unexplained cases. In this context, adenomyosis has progressively emerged as a biologically plausible and clinically relevant determinant, rather than a coincidental finding.

Adenomyosis is associated with profound structural and functional alterations of the endometrial–myometrial interface, which collectively compromise implantation and early pregnancy maintenance. At the molecular level, as previously reported, impaired decidualization represents a key feature, driven by progesterone resistance and altered expression of implantation-related genes, including those involved in cell adhesion, angiogenesis, and immune regulation. These abnormalities are embedded within a chronic inflammatory microenvironment characterized by increased cytokine production and altered immune cell infiltration, which may disrupt embryo–endometrial synchrony and impair early placental development [3,98,99]. In addition, aberrant expression of integrins and other receptivity markers has been described, further supporting the hypothesis of a dysfunctional endometrium.

Beyond molecular alterations, adenomyosis also induces biomechanical changes. Disruption of the JZ has been associated with abnormal uterine peristalsis and increased contractility, which may impair embryo transport, apposition, and stable implantation. These functional disturbances may be particularly relevant in the peri-implantation period, where subtle alterations in uterine dynamics can influence implantation success. Furthermore, early vascular remodeling may be compromised, predisposing to defective placentation and pregnancy instability.

The clinical relevance of these mechanisms is supported by a consistent body of evidence. Meta-analyses have demonstrated a significantly increased risk of miscarriage in women with adenomyosis, with similar trends observed across spontaneous conception and ART cycles [96,100]. Studies focusing on ART outcomes further report reduced clinical pregnancy and live birth rates, alongside increased early pregnancy loss [85,99].

Furthermore, a recent study suggests that internal adenomyosis may be more strongly associated with RPL compared with other disease phenotypes [7]. Importantly, recent large-scale analyses indicate that oocyte morphology and intrinsic embryo quality are largely preserved in adenomyosis, whereas miscarriage rates remain elevated, suggesting that the primary defect lies within the uterine environment rather than the embryo [101,102].

Nevertheless, the literature remains heterogeneous. Differences in diagnostic criteria, particularly between ultrasound- and MRI-based definitions, and the lack of standardized classification systems limit comparability across studies. In addition, the frequent coexistence of adenomyosis with endometriosis introduces potential confounding, as both conditions share overlapping pathophysiological pathways. Despite these limitations, the convergence of mechanistic, clinical, and ART-based evidence supports a model in which adenomyosis actively contributes to RPL, particularly in otherwise unexplained cases. This has important implications, suggesting that adenomyosis should be systematically considered in the diagnostic work-up and may represent a modifiable target in selected patients.

### 3.7. Maternal-Fetal Outcomes

The influence of adenomyosis extends beyond implantation and early pregnancy, affecting placentation and fetal development in a manner that translates into clinically relevant obstetric risk. Increasing evidence indicates that adenomyosis is associated with a spectrum of adverse maternal and foetal outcomes, supporting the concept that its impact persists throughout gestation.

Epidemiological data derived from meta-analyses and large observational studies consistently demonstrate increased risks of hypertensive disorders of pregnancy, preterm birth, cesarean delivery, fetal malpresentation, and postpartum hemorrhage in women with adenomyosis [96]. In parallel, several studies report higher rates of low birth weight and small-for-gestational-age infants, reinforcing the hypothesis of impaired uteroplacental function [95,99]. Notably, these associations appear to be largely independent of the mode of conception, suggesting that the underlying uterine pathology is a primary determinant rather than a secondary effect of assisted reproduction.

A recent systematic review and meta-analysis specifically addressing the independent effects of adenomyosis, endometriosis, and ART-related factors further identified associations between adenomyosis and miscarriage and pre-eclampsia, although the overall quality of evidence was considered low [103].

At the core of these complications lies defective placentation. Structural and functional alterations of the JZ may impair trophoblast invasion and spiral artery remodeling, leading to inadequate transformation of uterine vasculature and reduced uteroplacental perfusion [104]. Chronic inflammation and altered endocrine signaling further contribute to endothelial dysfunction and placental insufficiency, providing a coherent biological explanation for the increased risk of pre-eclampsia, fetal growth restriction, and preterm birth. In addition, abnormalities in uterine contractility may contribute to premature cervical changes and preterm labor.

Recent studies have provided further insight into the role of disease burden and phenotype. Increased adenomyosis volume has been associated with higher rates of preterm birth, placenta previa, and cervical incompetence, suggesting a dose–response relationship between disease severity and obstetric complications [105]. Moreover, phenotype-specific analyses indicate that diffuse adenomyosis and lesions located near the placental implantation site are associated with worse outcomes, including earlier delivery and increased postpartum hemorrhage [10,73].

For example, a recent single-center retrospective cohort study conducted on singleton pregnancies with a confirmed diagnosis of adenomyosis between 2014 and 2019 [73] demonstrated greater blood loss during delivery when the placenta was implanted over an adenomyotic lesion located in the inner myometrium. Regarding postpartum hemorrhage, the same study showed that postpartum blood loss was significantly higher in patients with diffuse adenomyosis compared with those presenting focal forms (internal or external). Furthermore, the same study highlighted that, in patients with inner myometrial adenomyosis, placental implantation directly overlying an adenomyotic lesion may represent an increased risk factor for preterm birth and, consequently, for reduced mean gestational age [73]. Additional observational evidence comes from a multicenter retrospective cohort study by Giorgi et al. [106], which reported a histologically confirmed prevalence of adenomyosis of 39.4% among women undergoing postpartum hysterectomy for postpartum hemorrhage.

However, inconsistencies across studies should be acknowledged. Some analyses report attenuated associations after adjustment for confounders, particularly in mild disease. Variability in diagnostic criteria, imaging modalities, and study design further contributes to heterogeneity. In addition, the frequent coexistence with endometriosis complicates the attribution of independent effects. Despite these limitations, the overall consistency of epidemiological and mechanistic evidence supports a clinically meaningful impact of adenomyosis on maternal–fetal outcomes. Importantly, the available data suggests that pregnancy risks may differ according to the anatomical phenotype of the disease. Available evidence suggests that obstetric risk may vary according to adenomyosis phenotype, although phenotype-specific associations remain insufficiently defined. Internal adenomyosis has been associated with placental dysfunction-related complications, including impaired placentation, foetal growth restriction, preterm birth, and reduced gestational age at delivery, whereas postpartum hemorrhage appears to be influenced by disease extent and placental localization rather than by external adenomyosis specifically [73,74]. This distinction mirrors the broader concept emerging throughout the literature, namely that inner and outer myometrial adenomyosis may represent biologically and clinically distinct entities with different implications for fertility, implantation, placentation, and obstetric outcomes.

From a clinical perspective, these findings justify a more individualized approach to pregnancy management in women with adenomyosis, considering not only the presence of the disease but also its phenotype and anatomical distribution. Risk stratification based on inner versus outer myometrial involvement may improve surveillance strategies and optimize maternal and fetal outcomes. To facilitate comparison of the clinical evidence supporting phenotype-, localizations-, and disease-burden-specific associations, the key primary human studies are summarized in Table 2.

## 4. Therapeutic Strategies

The clinical recognition of adenomyosis as an active determinant of reproductive dysfunction and adverse pregnancy outcomes necessitates a tailored therapeutic approach. Given the heterogeneous presentation of the disease, management strategies should be aligned with the specific reproductive endpoint, including infertility, recurrent pregnancy loss, and maternal–fetal outcomes.

### 4.1. Infertility

In the context of infertility, therapeutic strategies primarily aim to restore endometrial receptivity and optimize the uterine environment prior to conception. Hormonal suppression represents the cornerstone of medical management. Gonadotropin-releasing hormone agonists (GnRHa) are widely used to induce a hypoestrogenic state, resulting in regression of adenomyotic lesions and modulation of inflammatory pathways. Several studies suggest that prolonged GnRHa pretreatment before ART may improve implantation and clinical pregnancy rates, particularly in women with more severe disease; however, findings remain inconsistent across meta-analyses, reflecting variability in treatment protocols and patient selection [76,85].

More recently, GnRH antagonists have emerged as a potential therapeutic option for the management of adenomyosis-related symptoms owing to their rapid and reversible suppression of ovarian steroidogenesis; however, evidence regarding their impact on fertility and reproductive outcomes remains limited [34].

Beyond hormonal suppression, optimization of ART strategies has been proposed. Approaches such as extended downregulation protocols, freeze-all strategies, and deferred embryo transfer have been suggested to improve outcomes, although robust comparative evidence is lacking [107]. A recent meta-analysis comparing different IVF/ICSI treatment protocols suggested that treatment strategy may influence reproductive outcomes in women with adenomyosis, although the available evidence remains heterogeneous [108]. Notably, despite preserved oocyte morphology and embryo quality, women with adenomyosis consistently exhibit reduced clinical pregnancy and live birth rates, highlighting the central role of uterine factors in infertility [3,101,109].

Surgical intervention, particularly adenomyomectomy, may be considered in selected cases, especially in women with focal disease or significant uterine distortion. Potential benefits include restoration of uterine architecture and reduction in abnormal contractility. However, the evidence supporting improved fertility outcomes remains limited and largely observational, and must be balanced against surgical risks, including adhesion formation and potential impact on uterine integrity [85].

Alternative uterus-preserving approaches, such as myolysis, have also been proposed for selected patients; however, the available evidence regarding their impact on fertility outcomes remains scarce and is currently insufficient to support their routine use in reproductive medicine [110].

Overall, infertility management in adenomyosis remains largely empirical and should be individualized, considering disease phenotype, patient age, and prior reproductive history.

### 4.2. Recurrent Pregnancy Loss

In women with recurrent pregnancy loss, therapeutic strategies should primarily target the underlying uterine dysfunction associated with adenomyosis. Given the central role of impaired decidualization, chronic inflammation, and abnormal uterine peristalsis, interventions aimed at modulating the endometrial environment are of particular interest.

Hormonal suppression with GnRHa has been proposed as a strategy to improve endometrial receptivity and reduce inflammatory activity prior to conception. While some evidence suggests a potential benefit in improving implantation and reducing early pregnancy loss, data remain limited and heterogeneous, with no consensus on optimal treatment duration or protocol [76,85]. Similarly, the role of surgical treatment in RPL is not well defined, and current evidence does not support routine intervention outside selected cases.

Adjunctive approaches such as preimplantation genetic testing for aneuploidy (PGT-A) have been explored in RPL populations. Although meta-analyses suggest improved live birth rates per transfer, these findings primarily reflect the selection of euploid embryos and do not directly address the uterine contribution to pregnancy loss [111]. Given that adenomyosis predominantly affects the uterine environment, the benefit of embryo-focused strategies may be limited unless combined with interventions targeting uterine pathology.

Importantly, the recognition of adenomyosis as a contributor to RPL underscores the need for a shift in clinical perspective, from predominantly embryo-centered approaches toward integrated strategies addressing uterine dysfunction.

### 4.3. Maternal–Fetal Outcomes

In contrast to infertility and RPL, where therapeutic interventions are aimed at achieving and maintaining pregnancy, management of maternal–fetal outcomes in adenomyosis is primarily focused on risk mitigation during gestation.

Given the association between adenomyosis and placental dysfunction, hypertensive disorders, and preterm birth, early identification of affected patients is crucial. While no specific pharmacological therapy has been validated to prevent obstetric complications in this population, increased antenatal surveillance represents a key component of management. This may include closer monitoring of fetal growth, uteroplacental blood flow, and cervical length, particularly in patients with severe or diffuse disease [73,105].

Current evidence suggests that pregnancies complicated by adenomyosis may benefit from management within high-risk obstetric care pathways, owing to the increased incidence of placental dysfunction–related complications, preterm birth, and postpartum hemorrhage. Furthermore, although no preventive intervention has been specifically validated for adenomyosis, low-dose aspirin may be considered in women presenting additional risk factors for pre-eclampsia according to standard obstetric recommendations [112]. However, the absence of standardized classification systems limits the implementation of phenotype-driven care [76].

At present, preventive strategies remain largely extrapolated from general obstetric practice, and no targeted interventions have been specifically validated for adenomyosis-related pregnancy complications. This highlights a critical gap in the literature and underscores the need for prospective studies focusing on preventive and therapeutic strategies in this high-risk population.

## 5. Discussion

**Figure 1:**
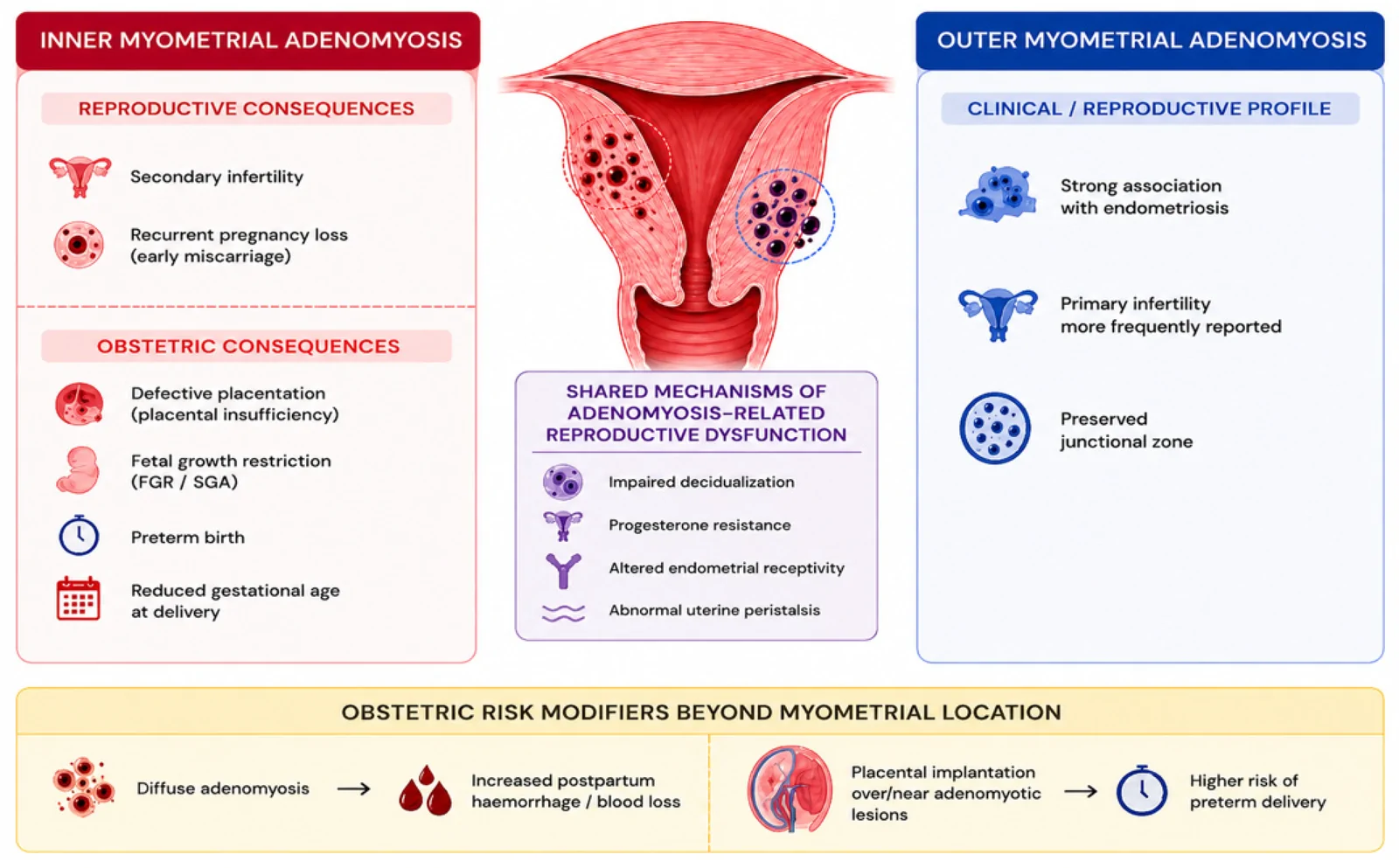
Phenotype-oriented model of adenomyosis showing the main reproductive and obstetric associations of internal and external disease, shared mechanisms of reproductive dysfunction, and additional obstetric risk modifiers related to diffuse disease and placental implantation over/near adenomyotic lesions. Phenotype-specific associations illustrated in this figure are based on emerging evidence and require further validation.

The findings compiled in this review reinforce the concept that adenomyosis plays a fundamental role in female reproductive failure and the development of obstetric pathologies. For decades, identifying the specific impact of adenomyosis on fertility has been hindered by its frequent co-existence with confounding factors such as endometriosis and uterine fibroids. However, contemporary literature provides robust evidence linking adenomyosis to compromised reproductive outcomes, spanning from spontaneous conception and embryonic implantation to gestation and delivery, as reported in Figure 1.

### 5.1. The Hostile Uterine Environment: Endometrial Receptivity and Uterine Peristalsis

Taken together, the mechanisms reviewed above suggest that adenomyosis-associated infertility should be interpreted as a complex uterine disorder rather than as the consequence of a single molecular or functional defect. Altered endometrial receptivity, dysregulated uterine contractility, progesterone resistance, and immune activation appear to converge on impaired embryo–endometrial synchrony and early placentation [90,91,92,93,94].

### 5.2. Redefining Recurrent Pregnancy Loss (RPL) and the Phenotype Paradigm

A particularly relevant concept emerging from recent evidence is that adenomyosis-related reproductive failure appears to be predominantly uterine rather than embryonic. Kahraman et al. [101], analyzing more than 200,000 oocytes, reported that adenomyosis does not significantly impair intrinsic oocyte quality, supporting the hypothesis that RPL and implantation failure primarily result from alterations of the uterine environment.

These findings further support the hypothesis that adenomyosis-related reproductive failure is largely driven by the uterine environment rather than by embryo competence [86,87].

Another major advance in the field is the recognition that adenomyosis should not be considered a homogeneous disease. Kolovos et al. [7] highlighted that different anatomical phenotypes are associated with distinct reproductive patterns. In particular, external adenomyosis is more frequently associated with primary infertility, likely because of its close relationship with pelvic endometriosis, whereas inner myometrial adenomyosis appears to be more strongly associated with secondary infertility and recurrent pregnancy loss, suggesting a direct effect of junctional zone involvement on early pregnancy maintenance.

These observations have important therapeutic implications. Rather than focusing exclusively on embryo quality, current evidence supports a more comprehensive reproductive strategy aimed at improving the uterine environment before conception. Selntigia et al. [76] emphasized the role of prolonged GnRH agonist pretreatment in reducing lesion activity and partially restoring endometrial receptivity, while Cozzolino et al. [85] demonstrated potential benefits for ART outcomes, although the available evidence remains heterogeneous.

At the same time, embryo-centered approaches should not be disregarded. Mumusoglu et al. [111] demonstrated that PGT-A may improve live birth rates in selected patients with unexplained recurrent pregnancy loss through the transfer of euploid embryos. However, this strategy does not correct the underlying uterine abnormalities associated with adenomyosis.

Overall, the available evidence supports a shift from a purely embryo-centered model of reproductive failure toward a more integrated and personalized approach that considers both embryonic competence and the uterine microenvironment. In this context, phenotype-oriented management may represent a promising strategy to optimize fertility and pregnancy outcomes in women with adenomyosis.

### 5.3. Placental Insufficiency and Late Obstetric Risk

The evidence reviewed above suggests that the clinical impact of adenomyosis may extend from implantation to placentation and delivery. From an interpretive perspective, the most relevant question is not only whether adenomyosis increases obstetric risk, but whether this risk varies according to disease burden, anatomical distribution, and the relationship between adenomyotic lesions and the placental implantation site [96,104]. This distinction may help explain the heterogeneity of published outcomes and provides a rationale for phenotype-oriented pregnancy surveillance, although the available evidence remains predominantly observational.

Recent studies have further demonstrated that obstetric risk may vary according to both disease burden and adenomyosis phenotype. Ni et al. [105], in a multicenter cohort study, reported that increasing adenomyosis volume is associated with higher rates of cervical incompetence, preterm birth, and other adverse perinatal outcomes, supporting a dose–response relationship between disease severity and pregnancy complications.

Phenotype-specific evidence has further refined this concept. Yang et al. [73] demonstrated that diffuse adenomyosis and lesions located beneath the placental implantation site within the inner myometrium are associated with reduced gestational age at delivery and increased postpartum hemorrhage. These findings suggest that the anatomical distribution of adenomyotic lesions may directly influence placental development and myometrial function during pregnancy.

Histopathological evidence from severe postpartum hemorrhage cohorts further supports the potential clinical relevance of adenomyosis in hemorrhagic obstetric complications [106].

Collectively, these observations support the emerging concept that obstetric risk may vary according to adenomyosis phenotype and anatomical distribution. Internal adenomyosis appears more frequently associated with placental dysfunction, fetal growth restriction, preterm birth, and reduced gestational age at delivery, whereas postpartum hemorrhage appears to be influenced by disease extent and placental localization rather than by external adenomyosis specifically [73,74]. Tsikouras et al. [6] further emphasized that this heterogeneity may have important implications for pregnancy management and maternal surveillance.

From a clinical perspective, these findings support a more individualized approach to pregnancy management in women with adenomyosis. Rather than considering adenomyosis as a single disease entity, phenotype-oriented risk stratification may help identify women at increased risk of placental dysfunction and obstetric complications, ultimately improving antenatal surveillance and maternal–fetal outcomes.

### 5.4. Adenomyosis and Endometriosis: Clinical Overlap and Management Implications

Adenomyosis and endometriosis frequently coexist, particularly in women with external adenomyosis [25,26,67,68]. Their clinical manifestations may substantially overlap, as both conditions can be associated with dysmenorrhea, chronic pelvic pain, dyspareunia, and infertility, complicating the attribution of individual symptoms and reproductive outcomes to either disease. In addition, shared inflammatory and uterine contractility-related mechanisms may contribute to their combined reproductive impact [94,95]. Comprehensive clinical and imaging assessment is therefore important to characterize both uterine adenomyosis and concomitant endometriotic disease and to avoid attributing reproductive failure to a single condition. The coexistence of endometriosis should also be considered when interpreting studies of adenomyosis, as it may confound estimates of the independent reproductive and obstetric effects of adenomyosis. At present, evidence supporting standardized combined treatment strategies remains limited; management should therefore be individualized according to the predominant symptoms, disease phenotype, reproductive goals, and extent of concomitant disease, ideally within an integrated multidisciplinary care pathway [95,113].

### 5.5. Strengths and Limitations

A strength of this narrative review is the integration of mechanistic and clinical evidence within a phenotype-oriented framework encompassing reproductive, obstetric, diagnostic, and therapeutic aspects of adenomyosis. Nevertheless, its narrative design does not provide the level of methodological standardization of a systematic review, and no formal risk-of-bias assessment was performed. Moreover, heterogeneity in diagnostic and phenotypic definitions, together with the predominantly observational nature of phenotype-specific evidence and potential confounding from coexisting gynecological conditions, limits the strength of definitive conclusions. These limitations should be considered when interpreting phenotype-specific associations and their potential clinical implications.

## 6. Future Perspectives

Adenomyosis is a highly heterogeneous condition characterized by a range of symptoms that do not present in the same way in all patients [114]. The ability to identify phenotype-specific profiles according to the type and anatomical location of the disease may improve counseling for young women and, at the same time, support individualized management strategies for infertility, recurrent pregnancy loss, and pregnancy care [6].

To date, although significant advances have been made in understanding the pathophysiology of adenomyosis, no therapies specifically targeting the underlying mechanisms of the disease are available. Therefore, appropriate management should focus on reducing the risk of infertility and obstetric complications potentially associated with the different disease phenotypes. In this regard, an accurate non-invasive diagnosis based on transvaginal ultrasound and magnetic resonance imaging, providing detailed information on disease type, location, and extent, is essential for appropriate patient management and reproductive counseling [13]. Further efforts toward standardized phenotype-oriented classification systems may improve the comparability of future studies and facilitate the development of tailored therapeutic and obstetric strategies [115]. Beyond pharmacological and surgical treatments, increasing attention is being paid to complementary and personalized interventions aimed at modulating chronic gynecological inflammation. Nutritional strategies have recently emerged as potential adjunctive approaches for the management of endometriosis-related symptoms and inflammation. Although evidence specific to adenomyosis remains limited, these interventions may represent a promising area for future research and could contribute to the development of personalized, phenotype-oriented management strategies [116].

Future research should focus on validating phenotype-specific therapeutic strategies through well-designed randomized clinical trials, with particular attention to reproductive outcomes and long-term disease control. Comparative head-to-head studies evaluating different hormonal treatments, including GnRH antagonists, are warranted to better define their relative efficacy, safety profile, tolerability, and impact on fertility preservation. In addition, further evidence is needed to establish the optimal duration of treatment, identify the most appropriate discontinuation strategies, and evaluate the risk of symptom recurrence following treatment withdrawal.

Another major challenge is the identification of reliable predictive biomarkers capable of stratifying patients according to disease phenotype and predicting treatment response, thereby supporting more individualized therapeutic decisions. Equally important is the validation of standardized phenotype-oriented classification systems integrating imaging features with clinical and molecular characteristics to improve patient stratification and guide personalized management. Advances in molecular profiling may further contribute to identifying novel therapeutic targets and refining precision medicine approaches. Longitudinal studies investigating disease progression and reproductive outcomes are also essential to better define the natural history of adenomyosis and optimize individualized care pathways.

## 7. Conclusions

Adenomyosis is a chronic condition with an early onset. Painful symptoms and abnormal uterine bleeding may appear at an early stage, and it is important to treat them to improve women’s quality of life. In these same patients, however, it is also important to assess the type of condition and its location in order to manage the risk of infertility and subsequent maternal-fetal complications during pregnancy appropriately. Accurate phenotypic characterization and integrating disease form, anatomical localization, and extent, may improve reproductive counseling and support more individualized therapeutic and obstetric management. Future studies using standardized diagnostic and classification criteria are needed to validate phenotype-specific risks and to develop targeted therapeutic strategies capable of improving reproductive and maternal–fetal outcomes.

## Figures and Tables

**Table 1:** Main molecular pathways involved in adenomyosis pathogenesis and potential therapeutic implications.

Pathway/Mechanism	Key Molecular and Cellular Players	Main Biological Effects	Potential Therapeutic Targets and Research Directions
**Hormonal dysregulation**	Estrogens, estrogen receptors, progesterone resistance	Increased proliferation, EMT, angiogenesis, myometrial invasion, impaired endometrial function	Optimization of hormonal therapies and patient stratification
**Inflammatory and immune dysregulation**	Macrophages, uterine NK cells, T cells; IL-6, TNF-α, IL-18, IL-10, TGF-β	Chronic inflammation, altered immune tolerance, fibrosis, impaired implantation	Identification of inflammatory targets and predictive biomarkers
**Tissue remodeling and fibrosis**	EMT pathways, extracellular matrix components, MMPs, TGF-β signaling	Junctional zone disruption, lesion invasion, abnormal uterine architecture	Development of anti-fibrotic and phenotype-oriented strategies
**Genetic and epigenetic alterations**	KRAS, PIK3CA, DNA methylation, histone modifications	Altered cell proliferation, differentiation, and hormonal signaling	Identification of molecular subtypes and novel therapeutic targets
**Cellular heterogeneity and molecular reprogramming**	Stromal fibroblast subsets, epithelial populations, immune clusters; single-cell RNA sequencing and spatial transcriptomics	Altered cell–cell communication, pathological remodeling, disease heterogeneity	Personalized medicine approaches integrating molecular, imaging, and clinical data

**Table 2:** Key primary human clinical studies evaluating phenotype-, localization-, and disease-burden-specific reproductive and obstetric outcomes in adenomyosis.

Study	Design and Population	Diagnosis/Phenotype Definition	Clinical Setting and Outcomes	Relevant Confounders/ Adjustment	Main Findings
**Shi et al., 2023** [10]	Retrospective cohort; 158 women aged 20–40 years undergoing fertility-sparing laparoscopic surgery; 103 provided detailed postoperative pregnancy data	Adenomyosis diagnosed by clinical assessment and 2D-TVUS and further verified by histology or MRI. TVUS localization: anterior (*n* = 32), posterior (*n* = 70), or both anterior and posterior (*n* = 56)	Postoperative spontaneous conception and IVF-ET; clinical pregnancy, pregnancy loss, and obstetric complications	The group with both anterior and posterior involvement had higher rates of ovarian endometrioma and pelvic adhesions and higher rAFS scores. No multivariable model specifically adjusting reproductive outcomes was reported	Cumulative pregnancy was significantly lower in women with both anterior and posterior involvement (log-rank *p* = 0.01). Severe obstetric complications were observed only in groups with posterior involvement, although differences in obstetric complications between groups were not statistically significant
**Bourdon et al., 2021** [67]	Observational cross-sectional study based on a prospectively managed surgical database; 248 women with MRI-diagnosed adenomyosis: isolated external (*n* = 109), isolated internal (*n* = 78), and combined phenotypes (*n* = 61)	MRI. Internal adenomyosis: JZmax ≥ 12 mm and JZmax/myometrial thickness >40%. External adenomyosis: lesion located in the outer myometrium, separated from an intact JZ by preserved normal myometrium	Women undergoing surgery for benign gynecological disease; infertility, HMB, pelvic pain, endometriosis, and clinical phenotype	Phenotype model adjusted for age, BMI, gravidity, previous uterine surgery, age at menarche, leiomyomas, and endometriosis. This model evaluated factors associated with phenotype and was not an infertility-outcome model	Infertility (34.0% vs. 20.5%) and primary infertility (25.1% vs. 9.0%) were more frequent with external than internal adenomyosis. External disease was strongly associated with endometriosis, particularly DE, whereas HMB was more frequent with internal disease. DE was independently associated with the external phenotype (aOR 27.34, 95% CI 5.24–142.66)
**Valdés-Bango et al., 2024** [68]	Cross-sectional study; 505 women with ultrasound-diagnosed adenomyosis: internal (*n* = 353) and external (*n* = 152)	TVUS. Internal: involvement of the JZ/inner myometrium with or without middle myometrium, without outer-myometrial involvement. External: involvement of the outer myometrium with or without middle myometrium, without JZ involvement	Tertiary endometriosis referral center; sonographic features, symptoms, infertility, DE, and leiomyomas	Multivariable phenotype model included age, nulliparity, number of adenomyosis criteria, DE, and myomas. Selection at a tertiary endometriosis center resulted in a high prevalence of concomitant endometriosis	Infertility did not differ significantly between internal and external phenotypes (45.3% vs. 48.7%; *p* = 0.488). External adenomyosis was independently associated with nulliparity (aOR 1.67) and DE (aOR 2.31), whereas leiomyomas were associated with internal adenomyosis
**Wang et al., 2025** [69]	Retrospective propensity-score-matched cohort; first FET cycle; 879 women with adenomyosis and 879 matched controls	TVUS using revised MUSA criteria. External: outer myometrium only; internal: inner/JZ and/or middle myometrium; mixed: both inner and outer myometrium	FET; live birth, implantation, pregnancy loss, and obstetric outcomes	1:1 propensity-score matching for age, BMI, AMH, infertility type, embryo stage, cause of infertility, FET protocol, and number of transferred embryos	Overall live birth was lower with adenomyosis than in controls (35.38% vs. 45.16%; OR 0.67). External adenomyosis showed a live-birth rate comparable with controls (45.80% vs. 46.85%), whereas live birth was lower with internal (27.44% vs. 42.33%; OR 0.52) and mixed disease (18.09% vs. 44.15%; OR 0.28). Early pregnancy loss was also increased overall
**Trinchant et al., 2025** [86]	Multicenter retrospective matched cohort; 228 women with adenomyosis and 228 controls undergoing single euploid embryo transfer; focal *n* = 62 and diffuse *n* = 166	TVUS; focal versus diffuse adenomyosis according to study sonographic criteria	Single euploid embryo transfer; implantation, miscarriage, live birth, and perinatal outcomes	Multivariable logistic regression corrected for age, BMI, smoking, endometriosis, fibroids, previous deliveries, and previous Cesarean sections	Live birth was lower with adenomyosis than in controls (24.6% vs. 46.9%). Within adenomyosis, live birth was lower with focal than diffuse disease (15.0% vs. 28.3%); after adjustment, focal disease remained associated with lower live birth (aOR 0.298, 95% CI 0.10–0.87) and implantation (aOR 0.41, 95% CI 0.17–0.94)
**Alson et al., 2024** [87]	Prospective cohort; 1037 women aged 25–≤39 years undergoing their first IVF/ICSI treatment	TVUS using revised MUSA definitions; direct and indirect features, including their localization in the inner/JZ or outer myometrium	First IVF/ICSI treatment; cumulative live birth and pregnancy loss	Modified Poisson models evaluated age, ovarian-reserve and treatment-related variables and endometriosis; main reported outcome aRRs were age-adjusted, with additional analyses stratified by endometriosis	Direct MUSA features were associated with lower cumulative live birth (24.5% vs. 42.7%; aRR 0.62, 95% CI 0.43–0.88) and higher miscarriage risk after FET (aRR 2.88, 95% CI 1.49–5.57). Features involving the inner/JZ were associated with markedly lower live birth (aRR 0.29, 95% CI 0.11–0.74); interrupted JZ showed the strongest individual association
**Iwasawa et al., 2021** [88]	Multicenter retrospective cohort; 52 women and 136 fresh or frozen-thawed ET cycles: advanced *n* = 40 (100 cycles), extrinsic *n* = 9 (27 cycles), intrinsic *n* = 3 (9 cycles)	MRI. Advanced: full-thickness myometrial involvement; extrinsic: lesion localized to the serosal side; intrinsic: lesion localized to the endometrial side	IVF/ICSI-ET; clinical pregnancy, pregnancy loss, live birth, and perinatal outcomes	Logistic regression adjusted for age, prior miscarriage, and BMI. Ovarian endometrioma was frequent in advanced and extrinsic disease; intrinsic subgroup was very small	Compared with advanced disease, extrinsic adenomyosis was associated with fewer pregnancy losses and more live births after adjustment; adjusted OR for live birth was 6.05 (95% CI 1.41–29.65). The intrinsic subgroup was too small for robust inference
**Sharma et al., 2026** [89]	Prospective cohort; 585 infertile women undergoing their first FET: 368 with diffuse adenomyosis (outer myometrium *n* = 167; JZ *n* = 201) and 217 male-infertility controls	TVUS using MUSA criteria; diffuse adenomyosis with ≥2 features, subsequently classified according to JZ or outer-myometrial localization	FET; pregnancy, clinical pregnancy, miscarriage, live birth, and pregnancy complications	Endometriosis was included as a comorbidity; observational design and limited subgroup size for pregnancy complications were acknowledged limitations	JZ involvement was associated with lower positive pregnancy (26.37% vs. 47.90%), clinical pregnancy (23.38% vs. 42.51%), and live birth (16.42% vs. 25.75%; OR 0.57, 95% CI 0.34–0.94) than outer-myometrial disease. Miscarriage did not differ significantly between the two adenomyosis locations
**Ni et al., 2024** [105]	Multicenter retrospective cohort; 321 singleton pregnancies with adenomyosis and 10,507 controls; adenomyosis volume available in 226 cases	TVUS or transabdominal ultrasound up to 14 weeks; disease burden assessed by adenomyosis volume. Severe adenomyosis defined as volume ≥757.5 cm^3^ (third quartile)	Maternal and perinatal outcomes, including PTB, pre-eclampsia, placenta previa, cervical incompetence, and fetal presentation	Adjusted for maternal age, parity, mode of conception, prior pregnancy losses <14 weeks, and prior pregnancy losses at 14–28 weeks	Adenomyosis was associated with increased PTB, placenta previa, cervical incompetence, and abnormal fetal presentation. Severe disease showed particularly high adjusted risks compared with controls, including PTB (aOR 5.50), pre-eclampsia (aOR 4.94), placenta previa (aOR 6.37), and cervical incompetence (aOR 12.79)
**Yang et al., 2026** [73]	Retrospective cohort; 255 women with adenomyosis who delivered: diffuse *n* = 119, intrinsic *n* = 89, and extrinsic *n* = 47; intrinsic disease further classified as lesion-attached *n* = 49 or lesion-unattached *n* = 40 according to placental location	Adenomyosis diagnosed by MRI or TVUS according to published criteria, or by typical findings at Cesarean section; study-specific diffuse, intrinsic, and extrinsic classification	PPH, blood loss, gestational age, preterm birth, and other maternal/perinatal outcomes	Multivariable logistic regression was used for key outcomes; major baseline characteristics, including fibroids and endometriosis, were comparable across phenotype groups	PPH and blood loss were higher in diffuse than focal phenotypes in unadjusted comparisons; after adjustment, adenomyosis phenotype itself was not an independent predictor of PPH, whereas placenta previa was. Within intrinsic disease, placental attachment over the lesion was associated with greater blood loss, lower gestational age (37.4 vs. 38.8 weeks), and more preterm birth (26.5% vs. 5.0%); placental attachment site independently predicted preterm delivery
**Qi et al., 2026** [74]	Retrospective cohort; 466 women with TVUS-diagnosed adenomyosis who achieved clinical pregnancy: internal *n* = 251 and non-internal *n* = 215	TVUS; internal versus non-internal adenomyosis according to lesion location	Natural or ART conception; live birth, miscarriage, PPROM, SGA, and other obstetric/neonatal outcomes	Multivariable analyses adjusted for age, gravidity, mode of conception, history of hysteroscopy, and ovarian endometriosis	Internal adenomyosis was associated with lower live birth (aOR 0.43, 95% CI 0.29–0.64) and higher early spontaneous miscarriage (aOR 2.27, 95% CI 1.29–4.10). Among live births, internal disease was associated with increased PPROM (aOR 3.06, 95% CI 1.37–7.27) and SGA (aOR 2.73, 95% CI 1.24–6.32)

## Data Availability

No new data were generated or analyzed in this study. All data discussed in this review are available in the cited publications.

## Abbreviations

2D, two-dimensional; 3D, three-dimensional; AMH, anti-Müllerian hormone; aOR, adjusted odds ratio; aRR, adjusted relative risk; ART, assisted reproductive technology; BMI, body mass index; CI, confidence interval; DE, deep endometriosis; DNA, deoxyribonucleic acid; EMT, epithelial–mesenchymal transition; ET, embryo transfer; FET, frozen embryo transfer; FGR, foetal growth restriction; GnRH, gonadotropin-releasing hormone; GnRHa, gonadotropin-releasing hormone agonist; HMB, heavy menstrual bleeding; ICSI, intracytoplasmic sperm injection; IL, interleukin; IVF, in vitro fertilization; JAK, Janus kinase; JZ, junctional zone; MeSH, Medical Subject Headings; MMPs, matrix metalloproteinases; MRI, magnetic resonance imaging; MUSA, Morphological Uterus Sonographic Assessment; NK, natural killer; OR, odds ratio; PGT-A, preimplantation genetic testing for aneuploidy; PPH, postpartum hemorrhage; PPROM, preterm premature rupture of membranes; PTB, preterm birth; RNA, ribonucleic acid; RPL, recurrent pregnancy loss; RR, relative risk; rAFS, revised American Fertility Society; SGA, small for gestational age; STAT, signal transducer and activator of transcription; TGF-β, transforming growth factor beta; TIAR, Tissue Injury and Repair; TNF, tumor necrosis factor; TNF-α, tumor necrosis factor alpha; TVUS, transvaginal ultrasound.
